# Hydrogen peroxide inducible clone-5 mediates reactive oxygen species signaling for hepatocellular carcinoma progression

**DOI:** 10.18632/oncotarget.5322

**Published:** 2015-09-22

**Authors:** Jia-Ru Wu, Chi-Tan Hu, Ren-In You, Siou-Mei Pan, Chuan-Chu Cheng, Ming-Che Lee, Chao-Chuan Wu, Yao-Jen Chang, Shu-Chuan Lin, Chang-Shan Chen, Teng-Yi Lin, Wen-Sheng Wu

**Affiliations:** ^1^ Institute of Medical Sciences, College of Medicine, Tzu Chi University, Hualien, Taiwan; ^2^ Department of Laboratory Medicine and Biotechnology, College of Medicine, Tzu Chi University, Hualien, Taiwan; ^3^ Research Centre for Hepatology, Department of Internal Medicine, Buddhist Tzu Chi General Hospital and Tzu Chi University, Hualien, Taiwan; ^4^ Department of Surgery, Buddhist Tzu Chi General Hospital, School of Medicine, Tzu Chi University, Hualien, Taiwan; ^5^ Department of Surgery, Taipei Tzu Chi Hospital, Buddhist Tzu Chi Medical Foundation, School of Medicine, Tzu Chi University, Hualien, Taiwan; ^6^ Department of Laboratory Medicine, Hualien Tzu Chi Hospital, Buddhist Tzu Chi Medical Foundation, Hualien, Taiwan

**Keywords:** HGF, paxillin, metastasis, JNK, migration

## Abstract

One of the signaling components involved in hepatocellular carcinoma (HCC) progression is the focal adhesion adaptor paxillin. Hydrogen peroxide inducible clone-5 (Hic-5), one of the paralogs of paxillin, exhibits many biological functions distinct from paxillin, but may cooperate with paxillin to trigger tumor progression. Screening of Hic-5 in 145 surgical HCCs demonstrated overexpression of Hic-5 correlated well with intra- and extra-hepatic metastasis. Hic-5 highly expressed in the patient derived HCCs with high motility such as HCC329 and HCC353 but not in the HCCs with low motility such as HCC340. Blockade of Hic-5 expression prevented constitutive migration of HCC329 and HCC353 and HGF-induced cell migration of HCC340. HCC329Hic-5(−), HCC353Hic-5(−), HCC372Hic-5(−), the HCCs stably depleted of Hic-5, exhibited reduced motility compared with each HCC expressing Scramble shRNA. Moreover, intra/extrahepatic metastasis of HCC329Hic-5(−) in SCID mice greatly decreased compared with HCC329Scramble. On the other hand, ectopic Hic-5 expression in HCC340 promoted its progression. Constitutive and HGF-induced Hic-5 expression in HCCs were suppressed by the reactive oxygen species (ROS) scavengers catalase and dithiotheritol and c-Jun N-terminal kinase (JNK) inhibitor SP600125. On the contrary, depletion of Hic-5 blocked constitutive and HGF-induced ROS generation and JNK phosphorylation in HCCs. Also, ectopic expression of Hic-5 enhanced ROS generation and JNK phosphorylation. These highlighted that Hic-5 plays a central role in the positive feedback ROS-JNK signal cascade. Finally, the Chinese herbal derived anti-HCC peptide LZ-8 suppressed constitutive Hic-5 expression and JNK phosphorylation. In conclusion, Hic-5 mediates ROS-JNK signaling and may serve as a therapeutic target for prevention of HCC progression.

## INTRODUCTION

Hepatocellular carcinoma (HCC) is one of the most common deadly cancers worldwide. The poor prognosis of HCC is due to high recurrence rate mainly caused by intrahepatic metastasis (about 80%) or extrahepatic metastasis (about 20%) [1]. Therefore, prevention of metastasis is essential for HCC management. To address the issue, the suitable targets within the molecular pathways leading to HCC metastasis are needed to be identified.

The tumor microenvironment of HCC contains a lot of metastatic factors including transforming growth factor β (TGFβ) [2] and hepatocyte growth factor (HGF) [3, 4], which are capable of triggering HCC metastasis. Paxillin, one of the adaptor molecules critical for integrating the focal adhesion signaling [5–7], is known to be involved in HCC progression triggered by HGF [8, 9], integrin engagement [10] or overexpression of P21-activated protein kinase [11].

Within the paxillin superfamily, Hic-5 is the most homologous to paxillin, with minor differences in the number of N-terminal LD domains [5]. However, many biochemical properties, regulatory mechanisms and molecular functions of Hic-5 are rather different from those of paxillin [12]. Upon integrin engagement or growth factor stimulation, paxillin becomes phosphorylated, primarily on Tyr 31 and 118 [5, 6] or Ser 178 [13], mediating signal transductions for cell spreading and motility. Although there are tyrosine phosphorylation sites on Hic-5 such as Y38, Y60 between LD1 and LD2 domain [for review 12, 33], the aforementioned phosphorylating events of paxillin (at Y31, 118) do not occur on Hic-5, due to the lack of cognate tyrosine/Ser residues. On the other hand, expression of Hic-5 but not paxillin can be stimulated by TGFβ [14] or ROS [15], required for its biological activation. In spite of these discrepancies, Hic-5 was also capable of triggering tumor progression as paxillin, although *via* distinct molecular pathways [16]. Moreover, Hic-5 may cooperate with paxillin to regulate metastasis of breast cancer [17]. However, the role of Hic-5 in HCC has not been clarified thus far.

In this study, we found Hic-5 could be a potential prognosis maker and therapeutic target for prevention of HCC. On the signaling level, Hic-5 mediates the sustained ROS-JNK signaling required for triggering HCC progression.

## RESULTS

### Detection of Hic-5 as a HCC progression marker

**Figure 1:**
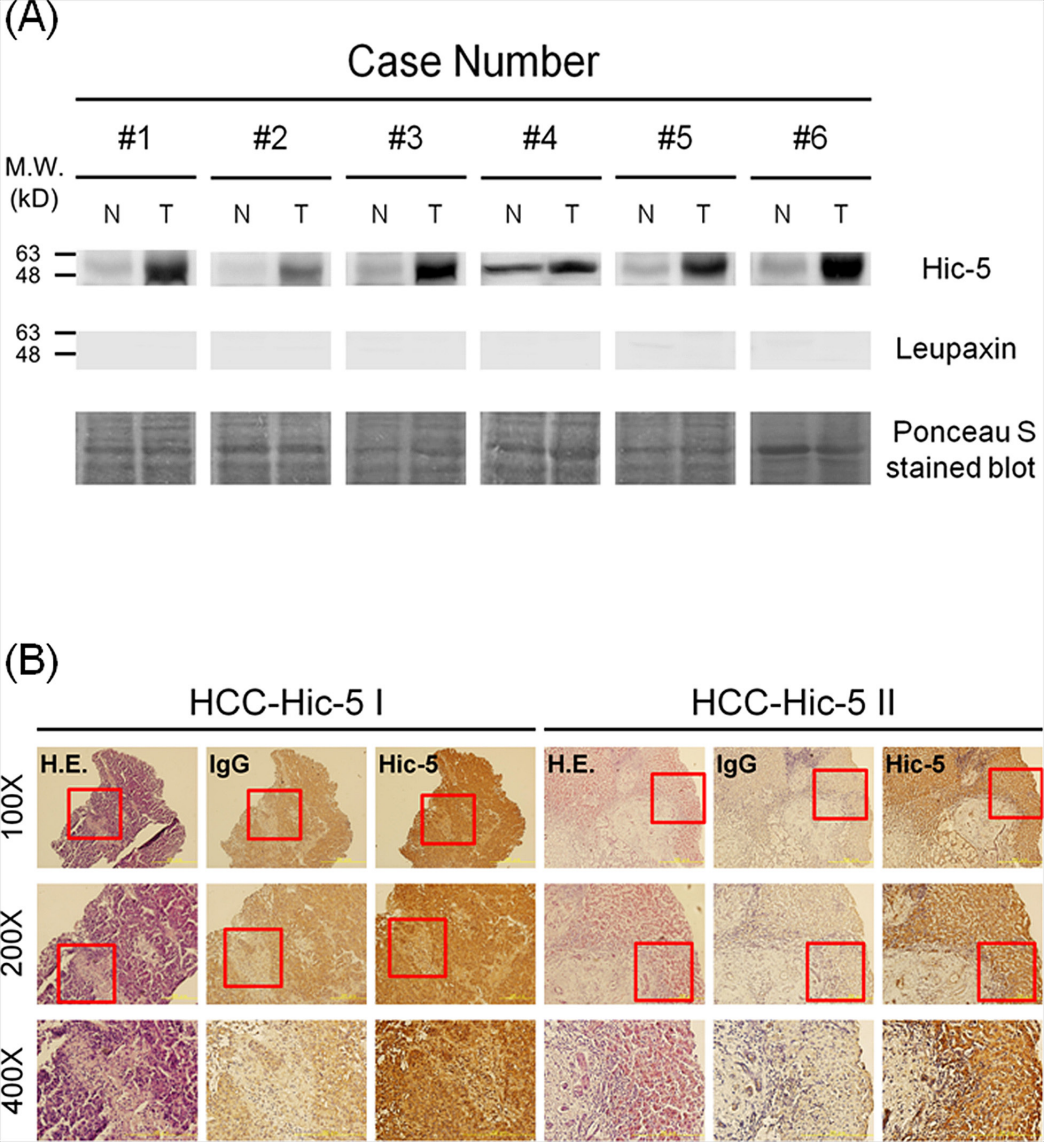
Detection of Hic-5 and Tyr31-phosphorylated paxillin in HCC tissues **(A)** Western blot of Hic-5 (upper panel) and leupaxin (middle panel) in tissue lysates of HCC from indicated patients using ponceau S stain as loading control. The data was representative of 3 reproducible experiments. N and T represent non-tumor and tumor sample respectively. The locations of indicated molecular weight marker are shown on the left. **(B)** IHC of Hic-5 coupled with IgG negative control and H & E stain were performed on the parallel tissue sections of 2 HCC cases. Red rectangle indicated the area within 100X field magnified to 200X and 400X shown below. The dark brown staining in Hic-5 IHC revealed the location of indicated molecule in contrast with the light brown staining of the same area in the negative IgG control.

Initially, we investigated whether Tyr31 phosphorylated paxillin [PXN (p-Y31)] can be a marker of HCC progression by examining the status of PXN(p-Y31) in HCC tissues obtained from surgery in TUZ CHI Hospital. In the pilot study using Western blot analysis, about 40.7% of the HCCs exhibited significant elevation of PXN(p-Y31) in tumor tissues, compared with that in the normal counterpart (Supplementary Figure S1, upper panel). Surprisingly, one more intensive band (around 50 kD) beneath PXN(p-Y31) (63–68 kD) can be observed in most of the HCCs screened as positive (Supplementary Figure S1, upper panel). We suspected this to be a protein belonging to the paxillin superfamily that strongly cross-reacted with the Ab against PXN(p-Y31). According to a previous report [5], Hic-5, one of the paxillin paralog with molecular weight most close to 50 kD, was suggested as the most possible candidate. Strikingly, Western blot of Hic-5 demonstrated dramatic elevation of Hic-5 in most of the HCCs with positive PXN(p-Y31) (Supplementary Figure S1, lower panel). To ascertain that the cross-reacted band was indeed Hic-5 but not other proteins related to paxillin, we further screened the expression of Hic-5 compared with leupaxin, another member of paxilin family with molecular weight slightly lower than 50 kD [5], in more HCC samples. As demonstrated in the Western blot of Hic-5 (Figure 1A upper panel), overexpression of Hic-5 (50 kD) can be observed in 6 HCCs but not the normal counterparts, whereas no band can be clearly observed in the same HCCs by Western blot of leupaxin (Figure 1A lower panel). Further, by screening 145 HCCs we found PXN(p-Y31) and Hic-5 increased in about 40.7% and 48.3% tissues respectively. Moreover, about 34.5% HCC tissues exhibited simultaneous elevation of both proteins (Supplementary Figure S2). Whether Hic-5 indeed expressed within the HCCs was examined *in situ*. As demonstrated in Figure 1B, IHC of Hic-5 on tissue sections of two Hic-5 overexpressing HCCs, denoted as HCC-Hic-5 I and HCC-Hic-5 II, revealed that Hic-5 was distributed within the tumor (but not non-tumor) region as indicated by hematoxylin/Eosin (H.E.) stain on the parallel tissue sections. In contrast, IHC using IgG as control revealed negative staining in the same region.

Further, the correlations of increased expression of Hic-5 and PXN(p-Y31) with metastatic potential of HCCs were analyzed. As shown in Table 1, 83.3%, 100% and 84.2% of HCCs with positive intrahepatic metastasis (I.M.), extrahepatic metastasis (E.M.) and total metastasis (T.M.), respectively, exhibited higher expression of Hic-5. In contrast, Hic-5 was higher in only 43.3%, 46% and 42.9% of HCCs with negative I.M., E.M. and T.M, respectively. Statistical analysis revealed that high Hic-5 expression in HCCs correlated well with I.M. (*p* = 0.001), E.M. (*p* = 0.01) and T.M. (*p* = 0.001) (Chi-square test, SPSS 16.0, *N* = 145) (Table 1). Also, the association of high Hic-5 with E.M. was confirmed by Fisher's Exact test (*p* = 0.011) (Table 1). In comparison, the correlations of PXN(p-Y31) with I.M. (*p* = 0.004) and T.M. (*p* = 0.008) were slightly poor than those of Hic-5 (Chi-square test, SPSS 16.0, *N* = 145) (Table 2). In addition, the correlations of PXN(p-Y31) with E.M. was insignificant (*p* = 0.186).

**Table 1:** Statistic analysis of the correlation of Hic-5 expression with m Metastatic potentials of HCC

Metastatic potentials	Hic-5			*P* value*
	All cases	Low expression (*T* < *N*)^#^	High expression (*T* > *N*)^&^	
**Intra-hepatic Metastasis**^a^				
*Positive*	18	3 (16.7%)^d^	15 (83.3%)	**0.001**
*Negative*	127	72 (56.7%)	55 (43.3%)	
**Extra-hepatic Metastasis**^b^				
*Positive*	6	0 (0%)	6 (100%)	**0.010 (0.011^%^)**
*Negative*	139	75 (54.0%)	64 (46.0%)	
**Total Metastasis**^c^				
*Positive*	19	3 (15.8%)	16 (84.2%)	**0.001**
*Negative*	126	72 (57.1%)	54 (42.9%)	

# Hic-5 expression in HCC tissue (T) lower than that in the normal counterpart (N) by over 1.5–2.0 fold (Note: the quantitation of Hic-5 expression in Table 1 & 2 were performed based on results of Western blot of Hic-5 in HCC tissues).

& Hic-5 expression in HCC tissue (T) higher than that in the normal counterpart (N) by over 1.5–2.0 fold.

* Statistical significance between high Hic-5 with indicated metastatic potential estimated by Chi-square test

% Statistical significance between high Hic-5 with indicated metastatic potential estimated by Fisher' Exact test

a Sites of intra-hepatic metastasis (I.M.) coupled with vascular invasion in liver, including microscopic vascular invasion in small vessels and macroscopic tumor invasion in bile duct, hepatic vein and portal vein can be observed.

b Extra-hepatic metastasis (E.M.) including regional lymph node metastasis and distant organ metastasis can be observed.

c Total Metastasis is equal to the summation of I.M. and E.M.

d The percentage in parenthesis represent the ratio of the number of HCCs with indicated Hic-5 expression status (i.e. *T* < *N* or *T* > *N*) *vs* total number of HCCs (all case) classified as either positive or negative of the indicated metastatic potential.

**Table 2:** Statistic analysis of the correlation of Tyr31- phosphorylated paxillin [PXN (p-Y31)] with Metastatic potentials of HCC

Metastatic potentials	PXN (p-Y31)			*P* value*
	All cases	Low expression (*T* < *N*)	High expression (*T* > *N*)	
**Intra-hepatic Metastasis**				
*Positive*	18	5 (27.8%)	13 (72.2%)	**0.004**
*Negative*	127	81 (63.8%)	46 (36.2%)	
**Extra-hepatic Metastasis**				
*Positive*	6	2 (33.3%)	4 (66.7%)	0.186
*Negative*	139	84 (60.4%)	55 (39.6%)	
**Total Metastasis**				
*Positive*	19	6 (31.6%)	13 (68.4%)	**0.008**
*Negative*	126	80 (63.5%)	46 (36.5%)	

# PXN (p-Y31) in HCC tissue (T) lower than that in the normal counterpart (N) by over 1.5–2.0 fold.

& PXN (p-Y31) in HCC tissue (T) higher than that in the normal counterpart (N) by over 1.5–2.0 fold.

* Statistical significance between high PXN (p-Y31) with indicated metastatic potential estimated by Chi-square test

a Sites of intra-hepatic metastasis (I.M.) coupled with vascular invasion in liver, including microscopic vascular invasion in small vessels and macroscopic tumor invasion in bile duct, hepatic vein and portal vein can be observed

b Extra-hepatic metastasis (E.M.) including regional lymph node metastasis and distant organ metastasis can be observed

c Total Metastasis is equal to the summation of I.M. and E.M.

d The percentage in parenthesis represent the ratio of the number of HCCs with indicated PXN (p-Y31) status (ie. *T* < *N* or *T* > *N*) *vs* total number of HCCs (all case) classified as either positive or negative of the indicated metastatic potential.

### Validating the role of Hic-5 in HCC progression using patient derived cell lines

**Figure 2:**
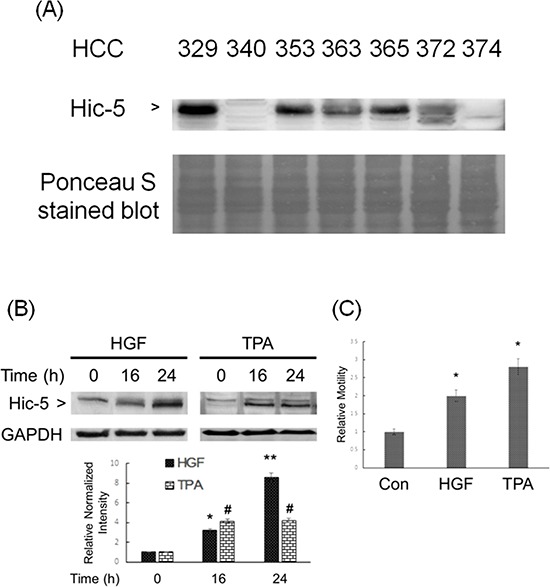
Constitutive and inducible expression of Hic-5 associated with motility of HCC Various patients derived HCC was untreated **(A)** HCC340 was treated with HGF (left panel) and TPA (right panel) for indicated time **(B)** and 48 h **(C)** Western blot of Hic-5 (A), (B) and wound healing motility assay (C) were performed. Nonspecific bands on Ponceau S stain blot (A) and GAPDH (B) were used as loading controls. In (B) and (C) the quantitative figures demonstrate the relative intensity ratio of Hic-5/GAPDH and relative motility, respectively, taking the data of time zero (B) and untreated sample (C) as 1.0. (**) and (*) (^#^) represent statistical significance (*p* < 0.005 and *p* < 0.05, respectively, *n* = 3) for differences between the indicated samples and time zero (B) or untreated group (C).

We further focused on investigating whether Hic-5 plays an essential role in the progression of HCC. Initially, we compared the expression of Hic-5 in several patient-derived HCC cell lines, the phenotypes of which such as cell motility have been characterized [20]. As shown in Figure 2A, Hic-5 markedly expressed in most of the HCCs such as HCC329, HCC353, HCC365 and HCC372 with average to high motility, slightly expressed in HCC363 with average motility but not in the non-motile HCC340 and HCC374 [20]. Notably, HCC329, the most motile HCC [20], expressed the highest Hic-5 among the HCCs examined.

We further investigated whether Hic-5 can be induced in the low Hic-5 expressing HCCs such as HCC340, H363 and HCC374 by metastatic factors such as HGF and the tumor promoter 12-O-tetradecanoyl-phorbol-13-acetate (TPA), known to be a potent inducer of HCC migration [19, 21, 22]. HepG2, the conventionally used HCC cell line with low motility and no constitutive Hic-5 expression as HCC340 (data not shown), was also included. As shown in Figure 2B, Hic-5 can be induced in HCC340 by 25 nM HGF to 3.8-fold at 16 h and increased to 8.0-fold at 24 h. Also, 50 nM TPA can induce Hic-5 expression to 4.0-fold at 16 h, which sustained until 24 h in HCC340. Consitently, both HGF and TPA can induce cell migration of HCC340 to 2.0 and 2.7-fold, respectively (Figure 2C). Similarly, HGF induced migration of HCC363 and HepG2 to 3.8- and 2.0-fold, respectively (Figure 5D), consistent with the HGF-induced Hic-5 expression in HepG2 and HCC363 (Supplementary Figure S3 and data not shown). However, HGF didn't induce Hic-5 expression in HCC374 at 24 h, although cell migration of HCC374 can be induced by HGF at 48 h (data not shown).

**Figure 3:**
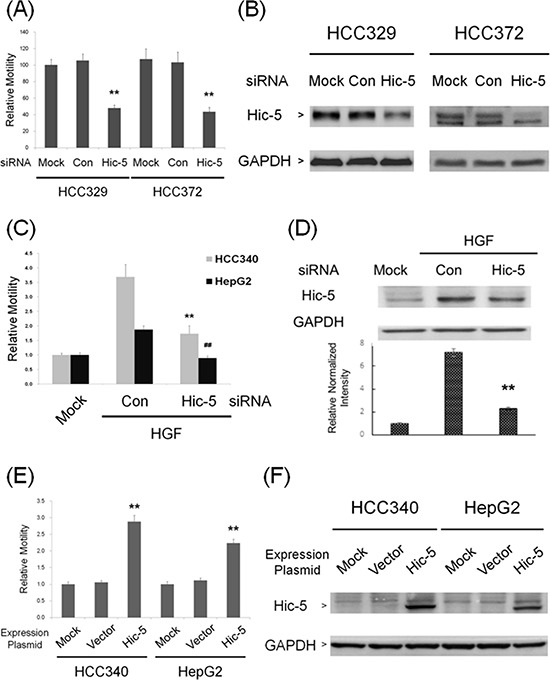
Hic-5 was required for constitutive and inducible HCC migration and sufficient for triggering HCC cell migration HCC 329 and HCC372 were untransfected (MOCK) or transfected with Hic-5 or control (Con) siRNA **(A, B)** HCC340 and HepG2 were untransfected (MOCK), transfected with indicated siRNA and treated with HGF for 24 h **(C, D)** HCC340 and HepG2 were untransfected (MOCK) or transfected with Hic-5 overexpressing plasmid TGFB1I1 **(E, F)** Wound healing motility assay (A, C, E) and Western blot of Hic-5 (B, D, F), were performed. In (A, C, E), relative motility was calculated, taking motility of MOCK as 1.0. In (B, D, F), GAPDH was used as a loading control for the Western blots. In (D), the quantitative figure is shown below. In (A, C, D, E), (**) and (^##^) represents statistical significance (*p* < 0.005, *n* = 3) for difference of relative migration (A, C, E) or relative intensity (D) between the indicated groups and the control siRNA (A, C, D) or vector (E) group. (B) and (F) are representatives of two reproducible results.

Whether Hic-5 was required for constitutive and inducible cell migration of HCC were investigated by transient RNA interference. As demonstrated in Figure 3A, motility of HCC329 and HCC372 greatly decreased after transfection with Hic-5 siRNA for 48 h by 60%, compared with the control siRNA group. The efficiency of Hic-5 siRNA was verified by the reduction of Hic-5 (by 85–92%) in both HCCs transfected with Hic-5 siRNA (Figure 3B). On the other hand, cell migration of HCC340 and HepG2 induced by HGF was decreased by prior transfection of the cells with Hic-5 siRNA by 73% and 100%, respectively, compared with the control siRNA group (Figure 3C). The efficiency of Hic-5 siRNA was validated by that HGF-induced Hic-5 expression in HCC340 and HepG2 transfected with Hic-5 siRNA decreased by 84% (Figure 3D) and 99% (Figure 7D), respectively, compared with the control siRNA group. To examine whether Hic-5 is sufficient for triggering cell migration of HCC, a Hic-5 cDNA plasmid, TGFB1I1, driven by the CMV promoter was employed. Remarkably, cell migration of HCC340, transfected with TGFB1I1 for 48 h increased to 3.0-fold, compared with the vector group (Figure 3E). Figure 3F demonstrated the dramatic increase of Hic-5 expression (to 7.5-fold) in cell transfected with TGFB1I1. Taken together, Hic-5 was essential for cell migration of HCCs.

**Figure 4:**
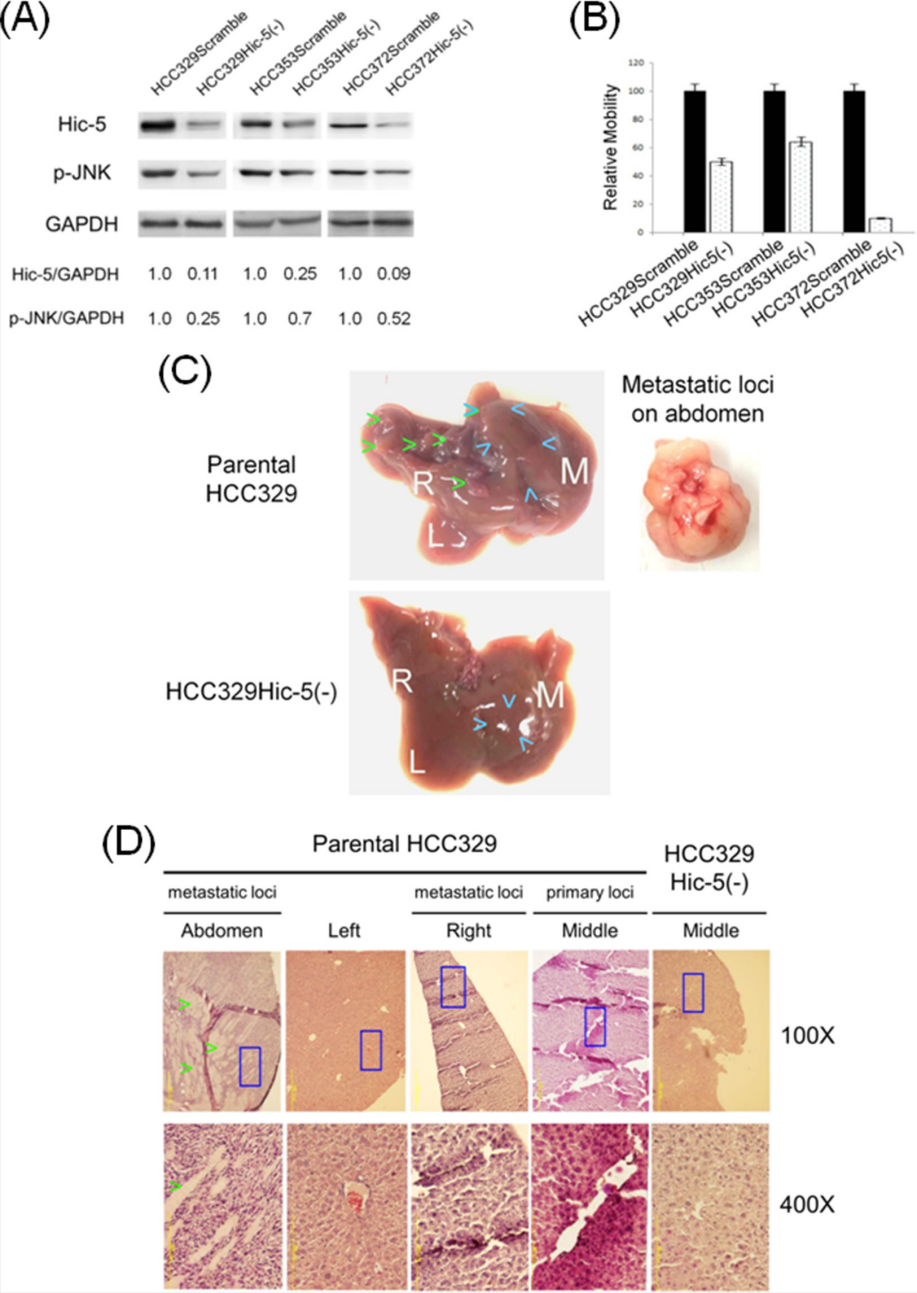
Depletion of Hic-5 suppressed JNK phosphorylation and progression of HCC329 Stable clone of HCCs expressing Scramble and Hic-5shRNA, denoted as HCC3xxScramble and HCC3xxHic-5(−), respectively, were obtained as indicated in Materials and Methods. Western blot of Hic-5 and phosphorylated JNK (p-JNK) **(A)** and cell migration **(B)** of indicated HCC3xxScramble and HCC3xxHic-5(−) were performed. In (A), GAPDH was used as a loading control. The numbers shown below were averaged (*N* = 2) relative intensity ratio of Hic-5/GAPDH p-JNK/GAPDH, taking the data of each HCC3xxScramble as 1.0. (B) is the quantitative figure of relative motility taking the data of each HCC3xxScramble as 1.0. The data shown are average from 2 reproducible experiments. **(C)** Pictures of whole liver of SCID mice sacrificed after injection of each of 20 × 10^5^ parental HCC329 (upper) and HCC329Hic-5(−) (lower) into middle lobe of liver for 2 months. The white letter M, L, and R represent middle, left and right liver lobes respectively. Blue and Green arrow heads indicated the location of primary and second tumors, respectively, in middle and right liver lobes. Tumor shown in the right panel was obtained from an extrahepatic metastatic lesion in abdomen. **(D)** H.E staining of normal tissue from HCC329Hic-5(−); primary and metastatic loci in indicated liver lobes (Middle, Right and Left) and extra metastatic loci on abdomen from parental HCC329. Blue rectangle indicates the area magnified from 100X to 400X. Green arrow head indicated the unidentified white-colored, longitudinal tissues in the extrahepatic metastatic lesion in abdomen.

To validate the role of Hic-5 in HCC progression, HCC329, HCC372 and HCC353 (the HCCs with high Hic-5) stably depleted of Hic-5, denoted as HCC329Hic-5(−), HCC372 Hic-5(−), and HCC353 Hic-5(−), respectively, were established by infection of the cells with a pseudoviral particle containing Hic-5 shRNA sequence inserted within a lentiviral vector. For comparison, each HCC was infected with the scramble shRNA to obtain HCC329Scramble, HCC372Scramble and HCC353Scramble, as negative controls. As demonstrated in Figure 4A, Hic-5 expression decreased in HCC329Hic-5(−), HCC372Hic-5(−), and HCC353Hic-5(−) by 89%, 91% and 75%, respectively, in comparison with that of each HCC expressing scramble shRNA. Consistently, motility of HCC329Hic-5(−), HCC372Hic-5(−), andHCC353Hic-5(−) greatly decreased by 52%, 91% and 35%, respectively, in comparison with that of each HCC3xxScramble (Figure 4B). Moreover, cell invasion assay using matri-gel coated cultured insert demonstrated that invasiveness of HCC329Hic-5(−) decreased by 50% compared with that of HCC329Scramble (Supplementary Figure S4). Similar result was observed in migration assay using cultured insert without matri-gel coating (Supplementary Figure S4). Thus, Hic-5 was essential for motility and invasiveness of the HCC. Further, the role of Hic-5 in metastatic capability of HCCs was investigated using the most motile HCC329 in a SCID mice model established previously [20]. The cells (as concentrated pellet) were injected into the middle lobe of liver followed by observation of the growth of primary tumor on middle lobe and metastatic loci on left or right lobe. As demonstrated in Figure 4C, HCC329 cells developed a substantial tumor in the middle lobe of liver two month after injection. Moreover, multiple loci of intra-hepatic metastasis (IM) can be observed on the right liver lobe, but with much less loci appearing on the left lobe. Surprisingly, a big white tumor was also observed on the abdomen (Fisher's exact test, SPSS *p* < 0.05, *N* = 3), indicative of extra-hepatic metastasis (Figure 4C). In contrast, in the mice inoculated with HCC329Hic-5(−), not only that the growth of primary tumor in the middle lobe of liver greatly decreased but also that no metastatic loci can be observed on both left and right lobe (Fisher's exact test, SPSS *p* < 0.05, *N* = 3) (Figure 4C). Moreover, HE staining of primary tumor (in the middle lobe) and metastatic lesion (in the right lobes) from mice inoculated with parental HCC329 showed characteristic hepatoma tissue in the form of trabeculae and cords with an irregular and dense tissue organization and deep stained nuclei (Figure 4D). Also, the extra-hepatic metastatic lesion on abdomen exhibited a more irregular and dense tissue organization composed of smaller tumor cells with deep stained nuclei. Surprisingly, a lot of unidentified white-colored, hard, longitudinal tissues were intercalated within the extra-hepatic metastatic tumor (Figure 4D). In contrast, very homogenous and regular architecture characteristics of the normal liver tissue were observed in the normal region of middle liver lobe from mice inoculated with HCC329Hic-5(−) or the normal region of left lobes from mice inoculated with parental HCC329 (Figure 4D). The requirement of Hic-5 for metastatic capability of HCC329 was validated by more than 6 reproducible animal experiments (Fisher's exact test, SPSS *p* < 0.05, *N* = 8), 3 of which are demonstrated in Supplementary Figure S5A. Remarkably, prominent intrahepatic and extrahepatic metastasis were observed in livers and abdomens, respectively, of mice injected with HCC329Scramble, in contrast with the dramatically reduced HCC progression in mice injected with HCC329Hic-5(−). Thus Hic-5 was required for tumor growth and intrahepatic/extrahepatic metastasis of HCC329 in SCID mice. In addition, metastatic capability of other non-Hic-5 expressing HCCs such as HCC340 and HCC374 was very low, compared with that of HCC329 (Supplementary Figure S5B).

### ROS signaling is essential for Hic-5 expression in HCCs

**Figure 5:**
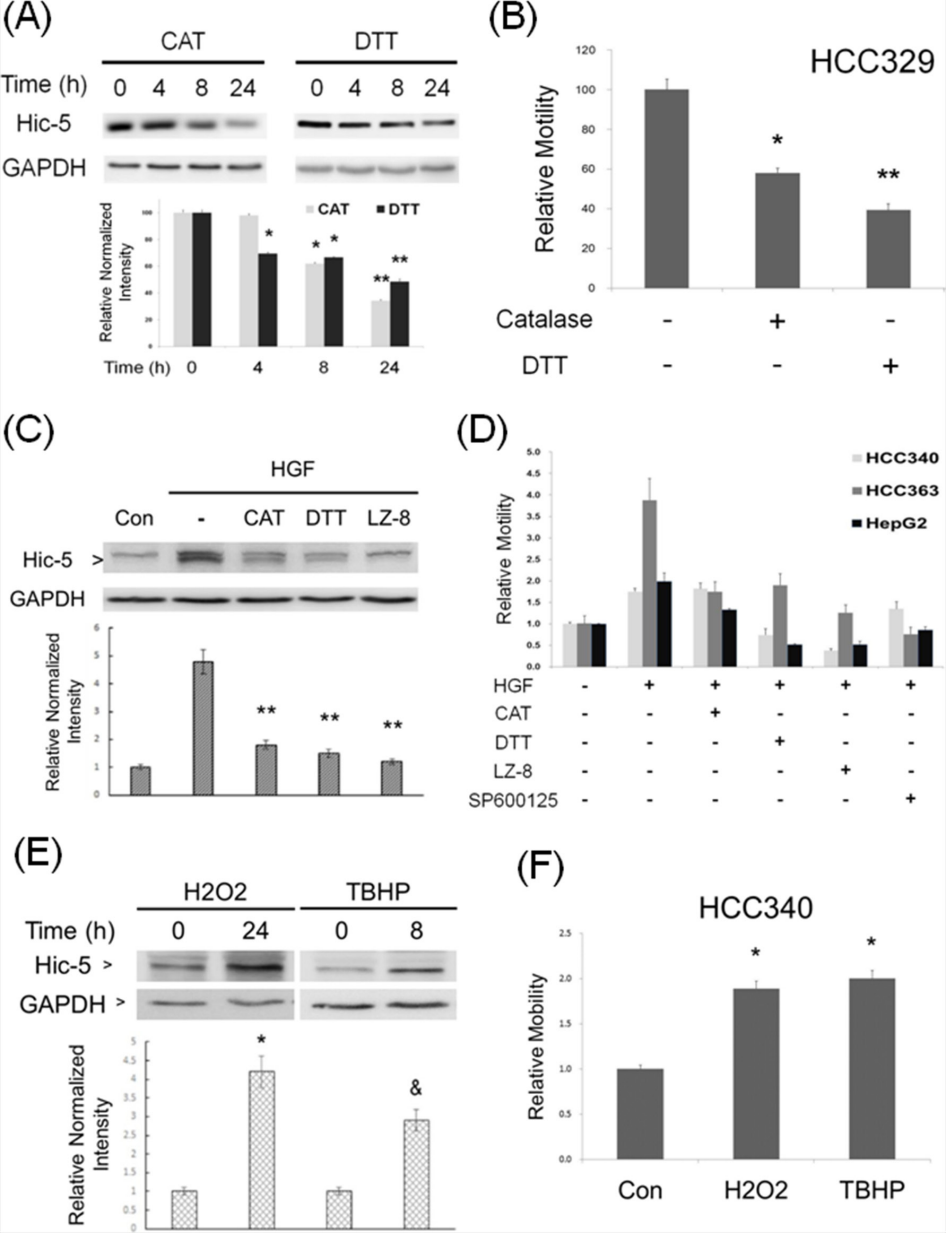
ROS is essential for constitutive and HGF-induced Hic-5 expression and HCC migration HCC329 was treated with 500 unit/ml catalase (CAT) or 0.5 mM dithiotheritol (DTT) for indicated times **(A)** or 48 h **(B)** HCC340 was untreated (Con), treated with HGF alone, or HGF coupled with the indicated inhibitors for 24 h **(C)** HCC340, HCC363 and HepG2 were untreated (−), treated with HGF alone, or HGF coupled with the indicated ROS scavengers, JNK inhibitor (SP600125, 20 μM) or LZ8 (2.5 μg/ml) for 48 h **(D)** HCC340 were treated with the indicated ROS generator for indicated times **(E, F)** Western blot of Hic-5 (A, C, E) and motility assay (B, D, F) were performed. GAPDH was used as loading control for the Western blots. The quantitative figures were demonstrated below each blot. In (B, D, F), relative motility was calculated, taking motility of the untreated sample as 100 (B) or 1.0 (D, F). (**) and (*) (^&^) represent statistical significance (*p* < 0.005 and *p* < 0.05, respectively, *n* = 4) for differences between the indicated samples and time zero (A, E), untreated (B, F) or HGF-only (C) groups. The data shown in (D) are average from 2 reproducible experiments.

We further investigated the mechanistic role of Hic-5 in HCC progression focusing on the signal pathway that Hic-5 involved. As its name indicated, Hic-5, the hydrogen peroxide inducible clone-5, was initially identified to be one of the clones in ROS-induced expression screen [15]. Moreover, ROS signaling was known to be essential for progression of a lot of tumors including HCC [23]. Therefore, we investigated whether Hic-5 expression is ROS-dependent. As demonstrated in Figure 5A, treatment of HCC329 with the H_2_O_2_ degradation enzyme catalase (CAT) (500 unit/ml) decreased Hic-5 expression by 15%, 35% and 62%, at 4, 8, and 24 h, respectively. Similarly, the antioxidant dithiotheritol (DTT) (0.5 mM) decreased Hic-5 expression by 30%, 60% and 75%, at 4, 8, and 24 h, respectively. Consistently, CAT and DTT suppressed cell migration of HCC329 by 40% and 60%, respectively (Figure 5B). Also, treatment of HCC353 and HCC372 with CAT and DTT for 24 h significantly decreased Hic-5 expression by 42–70% (Supplementary Figure S6A), consistent with the suppression of cell migration by 40–80% (Supplementary Figure S6B). Moreover, CAT and DTT blocked HGF-induced Hic-5 expression in HCC340 by 70% (Figure 5C), consistent with the suppression of HGF-induced HCC340 cell migration by CAT and DTT by 80–100% (Figure 5D). In addition, CAT and DTT also blocked HGF-induced cell migration of HCC363 and HepG2 by 30–50% and 50–80%, respectively (Figure 5D). ROS assay using DCF-DA labeling verified that treatment of CAT and DTT for 9–12 h decreased ROS generation by 45% and 80%, respectively in HCC329 (Supplementary Figure S7). Taken together, ROS was required for constitutive and HGF-induced Hic-5 expression and cell migration of HCCs. On the other hand, H_2_O_2_ and the superoxide (O_2_^−2^) generator tert-butyl hydroperoxide (TBHP) induced Hic-5 expression in HCC340 by 2.5 and 4-fold, respectively, (Figure 5E), consistent with the 2.0–2.2-fold increase of cell migration (Figure 5F). These results demonstrated that ROS was also sufficient for triggering Hic-5 expression and HCC cell migration.

### JNK is the downstream effector of ROS required for Hic-5 expression

**Figure 6:**
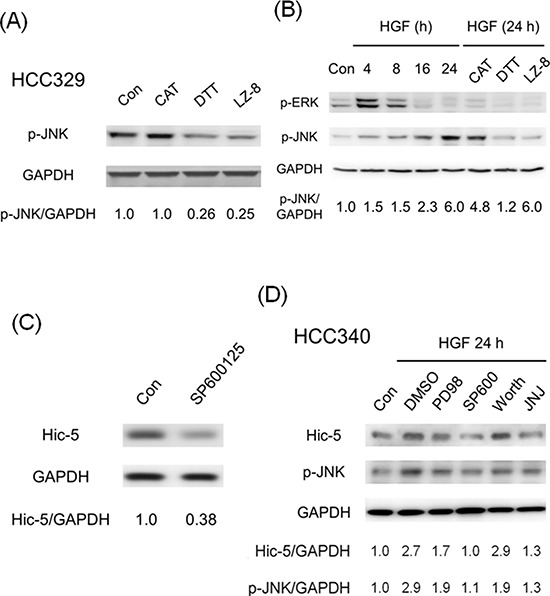
ROS-dependent JNK phosphorylation is required for Hic-5 expression in various HCCs HCC329 was treated with indicated ROS scavengers or LZ-8 (2.5 μg/ml) **(A)** or JNK inhibitor SP600125 (SP) (20 μM) for 24 h (C); HCC340 were untreated (Con), treated with HGF, or HGF coupled with indicated ROS scavenger or LZ-8 **(B)** or various inhibitors (D) for indicated time; Western blot of p-JNK (A), (B), (D), p-ERK (B) and Hic-5 **(C, D)** were performed using GAPDH as an internal control. The numbers shown below were averaged (*N* = 2) relative intensity ratio of p-JNK/GAPDH (A), (B), (D) and Hic-5/GAPDH (C), (D), taking the data of untreated (Con) as 1.0. In (D), PD98:PD98059, SP600: SP600125, Worth: Worthmannin, JNJ: JNJ38877605, a c-Met inhibitor.

In our previous studies, ROS-dependent activation of MAPK including Jun N-terminal kinase (JNK) and extracellular signal-regulated kinases (ERK), well known to mediate tumor progression [24, 25], were required for progression of HepG2 induced by TPA and HGF [8, 9, 19, 21, 22]. Moreover, JNK activation was required for constitutive migration of HCC329 [20]. Thus we investigated the relationship of ERK and JNK with ROS-Hic-5 cascade. As demonstrated in Figure 6A, constitutive JNK phosphorylation was suppressed by DTT in HCC329 at 8 h by 74% while CAT didn't exhibit suppressive effect as DTT at this time point. However, in a more extended time course study (Supplementary Figure S8), CAT significantly suppressed JNK phosphorylation at 24 h (but not at 8 h) by 55% in HCC329. This suggests that CAT had to take longer time than DTT for suppressing JNK phosphorylation in HCC329, probably due to the slower uptake of CAT. Moreover, CAT suppressed JNK phosphorylation at 8 h as DTT by 60–63% in HCC353, and the extent of inhibition exerted by both ROS scavengers was also higher (by 78–82%) at 24 h (Supplementary Figure S8). In contrast, constitutive ERK phosphorylation was only marginally observed in HCC329 and not influenced by both DTT and CAT (data not shown). The suppressive effect of both ROS scavengers on MAPK activation was further examined in HGF-treated HCCs. As demonstrated in Figure 6B, JNK phosphorylation gradually increased during HGF treatment from 4 to 24 h, with the maximal induction by 6.0 fold at 24 h in HCC340. Remarkably, the HGF-induced JNK phosphorylation at 24 h can be blocked by CAT and DTT by 25% and 90%, respectively (Figure 6B) in HCC340. Similarly, HGF-induced JNK phosphorylation at 24 h can be blocked by CAT and DTT by 40% and 95%, respectively in HepG2 (Supplementary Figure S9). On the other hand, HGF induced transient ERK phosphorylation during 4–8 h which decreased after 16–24 h, and was not influenced by CAT and DTT at 24 h (not shown). Notably, the extent of HGF-induced JNK phosphorylation during 16–24 h was the most prominent within the time course, coinciding with that of HGF-induced Hic-5 expression in HCC340 (Figure 2B). Since JNK was a well known downstream signal kinase responsible for transcriptional regulation, we investigated whether JNK activation is also required for Hic-5 expression. As was expected, the JNK inhibitor SP600125 (SP) suppress Hic-5 expression in HCC329 (Figure 6C), HCC372 and HCC353 (Supplementary Figure S6A, lane 4 in both panels) at 24 h by 62%, 85% and 79%, respectively. Moreover, HGF-induced Hic-5 expression in HCC340 (Figure 6D) and HepG2 (data not shown) can be totally suppressed by SP as efficiently as the c-Met inhibitor JNJ38877605. In contrast, it was slightly suppressed by PD98059, (the inhibitor of MEK, upstream kinase of ERK) and not influenced by worthmannin (the inhibitor of PI3-AKT) (Figure 6D). In addition, SP effectively prevented HGF-induced cell migration of HCC340, HCC363 and HepG2 (Figure 5D). Taken together, ROS-JNK cascade was required for both constitutive and HGF-induced Hic-5 expression.

### Hic-5 expression was essential for ROS generation and JNK phosphorylation

**Figure 7:**
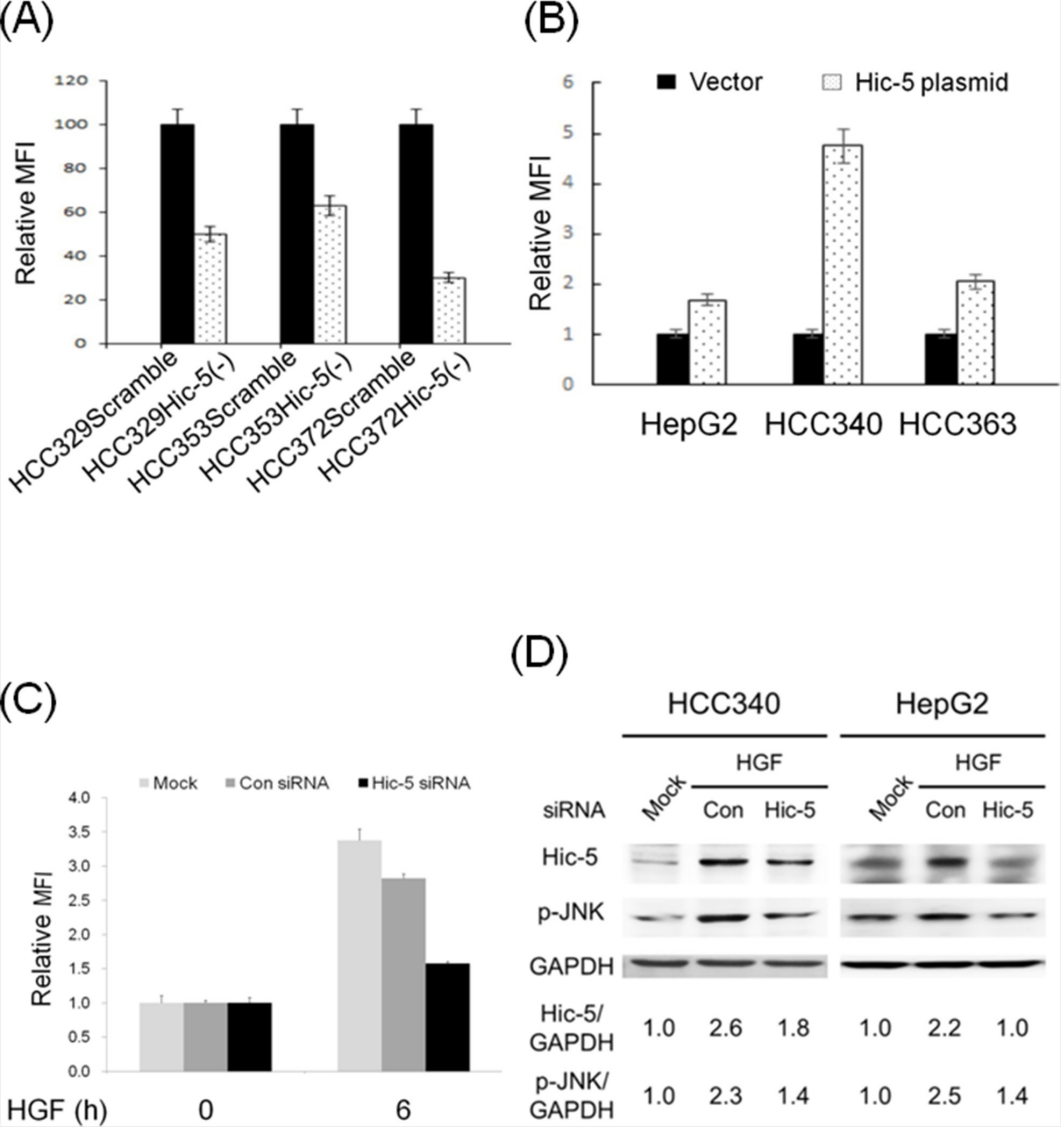
Hic-5 is required for constitutive and HGF-induced ROS generation and JNK phosphorylation, and sufficient for ROS generation and JNK phosphorylation HCCs stably expressing Scramble or Hic-5 shRNA as indicated were untreated **(A)** HCC340 and HepG2 were transiently transfected with Hic-5 overexpressing plasmid for 48 h **(B)** HepG2 were untransfected and untreated (MOCK), transiently transfected with control (Con) or Hic-5siRNA and treated with HGF for indicated time **(C)** HCC340 and HepG2 were untransfected (MOCK), transfected with Hic-5siRNA and treated with HGF for indicated time **(D)** ROS generation assay (A), B), and (C) and Western blot of Hic-5 and phosphorylated JNK (p-JNK) (D) were performed using GAPDH as an internal control. In (A), (B) and (C), MFI: mean fluorescence intensity representing the G mean of DCF fluorescence detected in flow cytometry. Relative MFI was calculated, taking HCCScramble (A), HCC transfected with vector (B) and control siRNA group (C) as 100 or 1.0 as indicated. The data shown are average from 2 reproducible experiments. In (D), the numbers shown below were averaged (*N* = 2) relative intensity ratio of p-JNK/GAPDH and Hic-5/GAPDH, taking the data of MOCK as 1.0.

One interesting study demonstrated that Hic-5 may directly associate with TRAF4/p47*phox* complex required for NADPH oxidase activation, and suggested a role of Hic-5 in triggering ROS generation in focal adhesion for cell migration [26]. Thus, it is tempting to observe whether Hic-5 was also essential for ROS generation in HCC. As demonstrated in Figure 7A, HCC329Hic-5(−), HCC372Hic-5(−) and HCC353Hic-5(−) exhibited decreased ROS generation by 30%, 70% and 35%, respectively, compared with each HCCScramble. On the other hand, transfection HepG2, HCC340 and HCC363 with Hic-5 expressing plasmid greatly increased ROS generation to 1.8, 4.9 and 2.2-fold (Figure 7B). In addition, blockade of Hic-5 expression by siRNA prevented HGF-induced ROS generation at 6 h in HepG2 (Figure 7C). Together, these indicated that Hic-5 also acted upstream of ROS. Since JNK is down stream of ROS, we further investigated whether Hic-5 was essential for JNK activation. As demonstrated in Figure 4A, phosphorylated JNK (p-JNK) greatly decreased in HCC329Hic-5(−), HCC372Hic-5(−) and HCC353Hic-5(−) by 75%, 48% and 30% respectively, compared with that in each HCCScramble. Moreover, blockade of Hic-5 expression by Hic-5 siRNA suppressed HGF-induced JNK phosphorylation at 24 h in HCC340 and HepG2 by 66% and 90%, respectively (Figure 7D). On the other hand, overexpression of Hic-5 significantly increased JNK phosphorylation to 2.1-fold in HCC340 (Supplementary Figure S10). Thus Hic-5 was also required for constitutive and HGF-induced JNK activation, and sufficient for triggering JNK activation in HCCs.

Collectively, ROS-JNK signaling can be both upstream and downstream of Hic-5, suggesting that Hic-5 may be responsible for establishing a positive feedback signal circuit for cell migration of HCC as elucidated in “Discussion”.

### LZ-8 suppressed Hic-5, p-JNK and ROS generation

**Figure 8:**
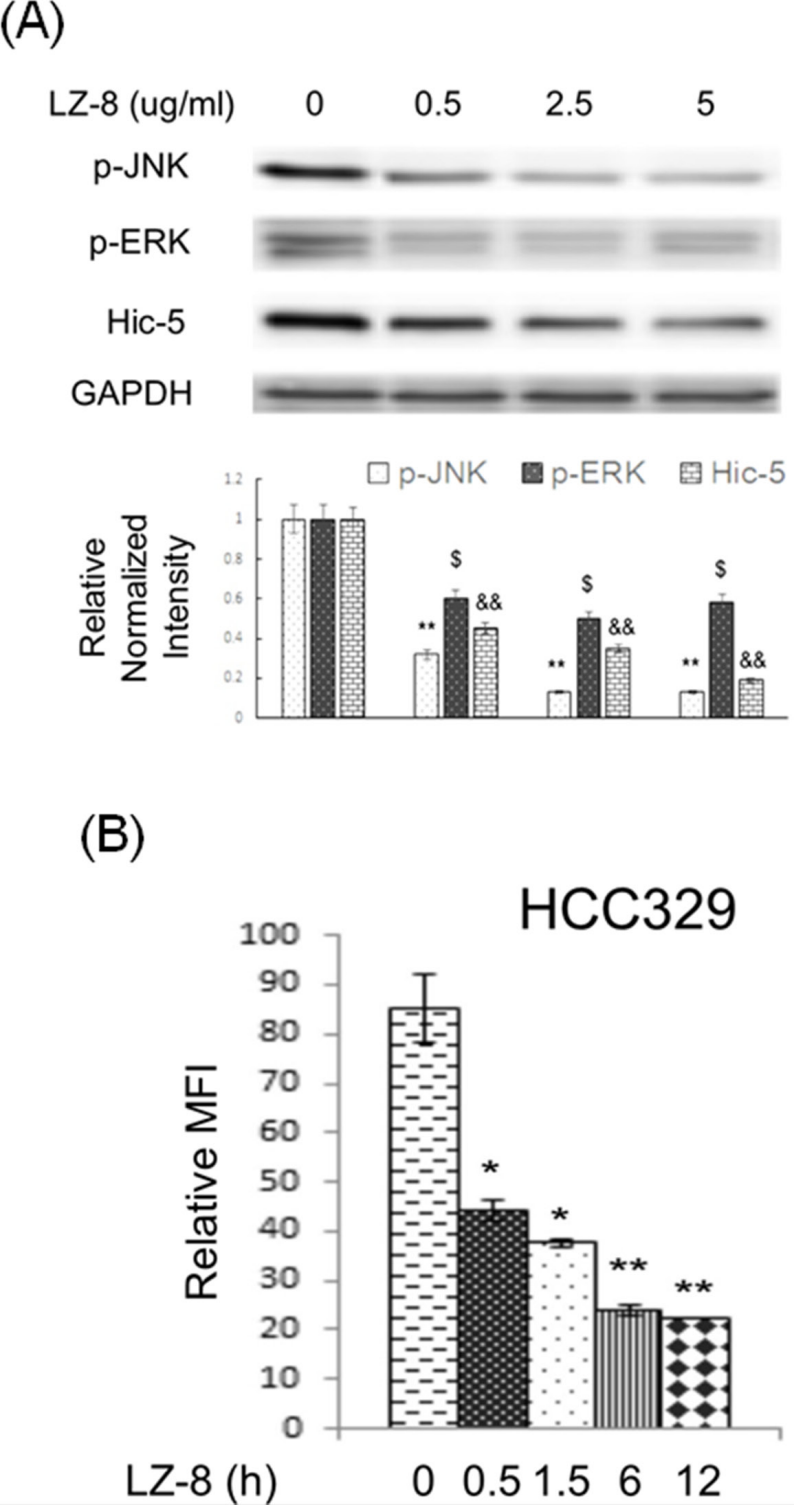
LZ-8 suppressed constitutive expression of Hic-5, JNK activation and ROS generation in HCC329 HCC329 were treated with LZ-8 at indicated concentration for 24 h **(A)** HCC329 were treated with 2.5 μg/ml LZ-8 at indicated time **(B)** Western blot of indicated molecule (A) and ROS assay (B) were performed. In (A), GAPDH were used as an internal control. Quantitative figures were demonstrated below. In (B), MFI: mean fluorescence intensity representing the G mean of DCF fluorescence detected in flow cytometry. (**) (^&&^) and (*) (^$^) represent statistical significance (*p* < 0.005 and *p* < 0.05, respectively, *n* = 4) for differences of intensity between the indicated groups and zero concentration (A) or time zero (B) group.

We further investigated whether the ROS-Hic-5-JNK pathway can be a potential therapeutic target for preventing HCC progression. Our recent report demonstrated that LZ-8 (also known as Lingzhi-8 or Reishi), a medicinal peptide purified from the Chinese herbal drug Ganoderma lucidium, prevented HCC progression of HCC329 *in vitro* and *in vivo* [20]. Interestingly, LZ-8 (at 0.5, 2.5 and 5.0 μg/ml) greatly suppressed constitutive Hic-5 expression and JNK phosphorylation in HCC329 by 85–60% in a dose dependent manner (Figure 8A). LZ-8 also decreased ERK phosphorylation in HCC329 by 40% at all concentrations. Consistently, treatment of 2.5 μM LZ-8 for 0.5, 1.5, 6 and 12 h suppressed constitutive ROS generation in HCC329 by 40–55% (Figure 8B). On the other hand, LZ-8 totally decreased the HGF-induced Hic-5 expression (Figure 5C), JNK phosphorylation (Figure 6B) and cell migration (Figure 5D) of HCC340. Interestingly, increased Hic-5 expression coupled with JNK phosphorylation in HCC340 transiently transfected with Hic-5 expressing vector can be totally suppressed by LZ-8 (Supplementary Figure S10). In addition, LZ-8 can also prevent the increased cell migration of HCC340 transiently transfected with Hic-5 expressing vector for 48 h (data not shown). Taken together, ROS-Hic-5-JNK signaling can be blocked by LZ-8 in HCC.

## DISCUSSION

### Hic-5 is a potential marker of HCC progression

Previous studies demonstrated that Hic-5 overexpressed in a varieties of invasive/metastatic cancers, including breast, lung, and prostate tumors and was emerging as a potential prognostic marker [16]. In the present study, we further demonstrated the direct correlation of Hic-5 with HCC metastasis in 145 HCCs (Table 1). The association of Hic-5 with HCC metastasis and the feasibility of Hic-5 as a HCC prognosis marker are worthy of further validation by screening more HCC samples.

### Hic-5 plays a critical role in HCC progression

The involvement of Hic-5 in tumor progression has been mentioned previously. Hic-5 expression can be induced by TGFβ leading to epithelial mesenchymal transition (EMT), cell migration, and invasion [27]. Furthermore, ectopic expression of Hic-5 is sufficient to promote normal mammary cells to undergo EMT [27]. In addition, the melanoma cell depleted of Hic-5 exhibit decreased cell motility and metastatic activity *in vivo* [28]. One recent report demonstrated the upregulation of Hic-5 in HCCs overexpressing proline-rich tyrosine kinase 2 (Pyk2) [29], which is known to be involved in HCC metastasis [30, 31]. Also TGF-β, which is responsible for triggering HCC progression [32, 33], can induce Hic-5 expression for malignant transformation [27]. In the present study, we further demonstrated that Hic-5 is essential for cell migration (Figure 3) and metastasis (Figure 4) of HCC.

### Hic-5 mediates positive feedback ROS-JNK signaling

**Figure 9:**
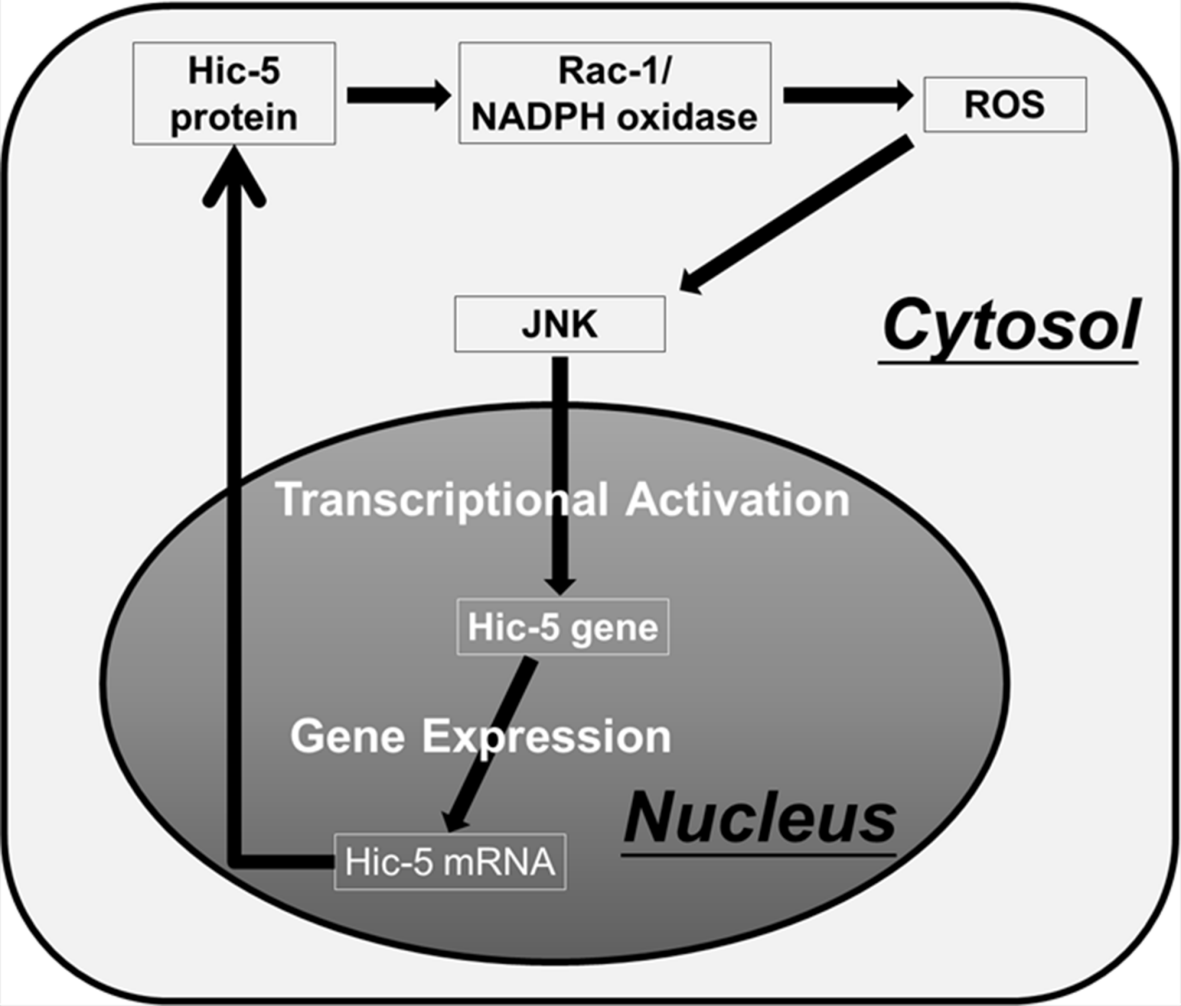
Proposed model for Hic-5-mediated sustained ROS-JNK signaling Based on inhibitor studies, ROS generation and JNK phosphorylation were required for constitutive and HGF-induced Hic-5 expression (Figure 5–6). Also, ectopic Hic-5 expression was sufficient for triggering ROS generation (Figure 7B) and JNK phosphorylation (Supplementary Figure S10). Taken together, Hic-5 can be both upstream and down of ROS-JNK. It can be proposed that the ROS triggered-JNK activation can induce transcriptional upregulation of Hic-5. The Hic-5 protein induced in turn activates Rac-1/NADPH oxidase dependent ROS generation [26] and JNK phosphorylation thus sustaining the signal transduction and Hic-5 expression. Accordingly, Hic-5 may play a central role in mediating the positive feedback ROS-JNK signaling circuit.

The diverse roles of Hic-5 in regulating signal transduction in various systems has been intensively studied [for reviews, 12, 16]. Previously, Hic-5 was found to promote TGFβ-induced signaling by binding to and inactivating the inhibitory Smads, Smad3 [34] and Smad7 [35] leading to enhanced TGF-β/Smad2/MAPK signaling required for EMT. Hic-5 also served as a scaffold protein that specifically activates the MAPK cascade [36]. Also, ROS signaling is capable of triggering Hic-5 gene expression [15] and nuclear translocation [37]. It was well established that ROS was essential for triggering tumor progression via a lot of signal cascades including MAPK [for review, 38]. For example, ROS may activate JNK to trigger EMT [39] known to be a critical step for tumor metastasis. One recent study demonstrated Nox2-dependent ROS-JNK signaling is essential for HGF-induced mobilization of endothelial progenitor cells (EPCs), involved in proangiogenesis and tumor progression [40]. In the present study, we found Hic-5 expression is closely related with ROS-JNK signaling during HCC progression. On the one hand, ROS generation and JNK phosphorylation were required for constitutive and HGF-induced Hic-5 expression (Figure 5, 6). On the other hand, not only that Hic-5 expression is required for constitutive and HGF-induced ROS generation (Figure 7A, 7C) but also that ectopic Hic-5 expression was sufficient for triggering ROS generation (Figure 7B) and JNK phosphorylation (Supplementary Figure S10). Taken together, Hic-5 can be both upstream and down of ROS-JNK, suggesting it plays the central role in establishing a positive feedback and sustained ROS and JNK signaling (see Scheme in Figure 9). The ROS and JNK signaling are known to be closely associated with sustained signal transduction for a lot of patho-physiological processes. For example, ROS are responsible for signal cross talks that triggers sustained MAPK activation and cell migration [41]. Also, ROS is critical for positive feedback loop triggering the sustained activation of Akt, which lead to mesangial cell hypertrophy and diabetic nephropathy [42]. On the other hand, sustained JNK signaling was required for Cylindromatosis (CYLD) (a deubiquitination enzyme) induced c-MYC expression and histone H3 methylations for HCC progression [43]. The detailed mechanism for Hic-5 to mediate sustained ROS-JNK signaling and HCC progression is worthy of further investigation.

### Hic-5 is a more ideal target for prevention of tumor progression

According to previous studies, both Hic-5 and paxillin are promising therapeutic targets for anti-HCC progression. However, target therapy aiming at paxillin seems unfeasible due to its ubiquitous tissue expression playing essential role for diverse biological functions [16]. In contrast, Hic-5 is enriched only in certain tissue such as smooth muscle and large intestine [16], thus may be a more suitable therapeutic target.

### LZ-8 suppressed HCC progression via blocking ROS-Hic-5-JNK signaling

In previous studies, LZ-8 was found to be both an immunomodulatory [44] and anti-tumor agent [45, 46]. On the signal level, LZ-8 may suppress the protein kinase C-dependent pathway [47] known to be involved in HCC progression triggered by HGF-c-Met [8]. Recently, we also found LZ-8 suppress HCC progression via blocking c-Met or c-Met-independent MAPK signaling [20]. In this study, we further found LZ-8 suppressed constitutive and HGF-induced expression of Hic-5 coupled with decrease of reactive oxygen species (ROS) generation and phosphorylation of JNK and ERK in HCCs. The detailed mechanisms for how LZ-8 suppressed ROS generation that block Hic5-MAPK signaling is worthy of further investigation.

In conclusion, we found the paxillin paralog Hic-5 plays an essential role in signal transduction for HCC progression and may serve as a promising prognosis marker and therapeutic target for management of HCC.

## MATERIALS AND METHODS

### Cell lines, hepatocellular carcinoma tissue collection, plasmid and chemicals

Human hepatoma cell HepG2 was purchased from the Bioresource Collection and Research Center (Hsinchu, Taiwan). HCC tissues were collected during HCC surgery at Tzu Chi Hospital with patient's consents, approved by the Research Ethics Committee in Buddhist Tzu Chi General Hospital (IRB 101-62). The tissues were snap frozen at −80°C before being harvested for Western blotting or sectioning for immunohistochemical analysis. HGF was obtained from Peprotech (Rocky Hill, NJ, USA). TPA, dithiotheritol, catalase and SP600125, PD98059 and JNJ38877605 were from Sigma (Milwaukee, DC, USA). Antibodies for Hic-5, phosphorylated JNK (p-JNK), phosphorylated ERK (p-ERK) and GAPDH were obtained from Santa Cruz Biotechnology, Inc. (California, USA). Antibody for Hic-5 was also obtained from GeneTex (Irvine, CA, USA). Hic-5 expression plasmid (TGFB 1I1) was from OriGene Technologies, Inc. (Washington, DC, USA). LZ-8 was from Yeastern Biotech Co., Ltd. (Taipei, Taiwan).

### Establishing patient-derived hepatocellular carcinoma cell lines

Clinically derived HCC cell lines were established from parts of HCC tissues obtained from surgery with patient's consents, approved by Buddhist Tzu Chi general hospital Research Ethic Committee (IRB 101-62). Briefly, HCC tissues were pretreated with collagenase and cultivated on the mitomycin C-treated NIH3T3 feeder layer for 4 to 6 passages to select the HCC cell lines. Homogenous HCC cell populations with the sustained proliferation ability (over 20 passages) were obtained. The characteristics of the HCC tumor cell lines were validated by detecting HCC tumor makers, such as Glypican 3 (GPC3) [18], after more than 40 passages.

### Immunohistochemistry

Immunohistochemistry (IHC) for Hic-5 was performed according to the standard protocols established by the Research Centre for Hepatology at Tzu Chi Hospital.

### Wound healing migration assay

Wound healing migration assay were performed according to our previous studies [8, 9]. Quantitation of cell motility was performed by counting the cells that have migrated into the blanking area using Image J software.

### Transwell migration/invasion assay

Cells were seeded on a 24-well transwell migration insert with (for invasion) or without (for migration) matrigel coating (Nalge Nunc International, Rochester, NY, USA) in a complete medium for 24 h. After appropriate treatments, cells that had migrated or invaded through the matrigel to the underside of the insert membrane were stained with 0.3% crystal violet. The cells on the topside of the insert membrane were rubbed with a cotton swab. The migrated/invaded cells on the underside were imaged using phase contrast microscopy with 200X magnification.

### Western blot

Western blots were performed according to our previous studies [8, 9]. The band intensities on the blots were quantified using Image J software.

### Flow cytometric analysis for ROS generation

ROS assay was performed as described in our previous report [19]. Each determination is based on the mean fluorescence intensity (MFI) of 5,000 cells.

### RNA interference and establishment of cells stably depleted of Hic-5

Hic-5 expression was transiently knocked down by transfection of the cells with 25 nM Hic-5 siRNA (Thermo Scientific, Dharmacon, US) for 48 h, according to the manufacture's protocol. To obtain stable clones expressing Hic-5 shRNA, lentiviral plasmids encoding shRNA for Hic-5 were packaged into 293T cell. Subsequently, the viral particle-containing mediums were used to infect HCCs followed by selection with puromycin for 2–3 weeks.

### Establishing hepatocellular carcinoma metastasis in SCID mice

The metastasis of HCC was established using Nod SCID mice as previously [8, 9]. All the mice were males, 8 weeks old, and had an average body weight of 35–40 g HCC cells (2 × 10^6^) were suspended in 100 μl DMEM and directly injected into the subserosa of the middle liver lobe under anesthesia. Two to four months after inoculation, the mice were sacrificed for examining the primary tumor growth on middle liver lobe and secondary tumor foci on left and right lobes. Nodules with diameters exceeding 0.1 to 0.2 cm on the left or right lobes were denoted as secondary tumor foci. Intrahepatic metastasis was defined if a minimum of two secondary tumor foci can be observed in the left and/or right liver lobes. Extrahepatic metastasis was defined by tumors appearing in organs other than the liver. During animal experiment, which was approved by the Institutional Animal Care and Use Committee at Tzu Chi University (No. 102080), regulations relevant to the care and use of laboratory animals were followed.

### Statistical analysis

Anova test was conducted to analyze the intensity differences between samples on the Western blot and the differences in cell motility between the indicated HCCs. Quantitative data were expressed as mean ± coefficient variation (CV), indicated by the error bars in each figure. The correlation of indicated molecules with HCC metastasis was analyzed by Chi-square test (SPSS 16.0 software, Chicago, IL, USA). The differences of HCC progression between mice inoculated with various HCCs were analyzed by Fisher's exact test (SPSS 16.0 software, Chicago, IL, USA).

## SUPPLEMENTARY FIGURES



## Abbreviations

Hic-5: Hydrogen peroxide inducible clone-5
ROS: reactive oxygen species
DTT: dithiotheritol
CAT: catalase
HGF: hepatocyte growth factor
IHC: Immunohistochemistry
PXN (p-Y31): Tyr31 phosphorylated paxillin
I.M.: intrahepatic metastasis
E.M.: extrahepatic metastasis
JNK: Jun N-terminal kinase
ERK: extracellular signal-regulated kinases
TPA: 12-O-tetradecanoyl-phorbol-13-acetate
LZ-8: Lingzhi-8
