# Supplementary material for: Hydrogen peroxide inducible clone-5 mediates reactive oxygen species signaling for hepatocellular carcinoma progression
Source: Oncotarget. 2015 Sep 22;6(32):32526–44. doi: 10.18632/oncotarget.5322 (PMC4741710; doi:10.18632/oncotarget.5322)
Supplement: Supplementary file 1 [file oncotarget-06-32526-s001.pdf]

SUPPLEMENTARY FIGURES

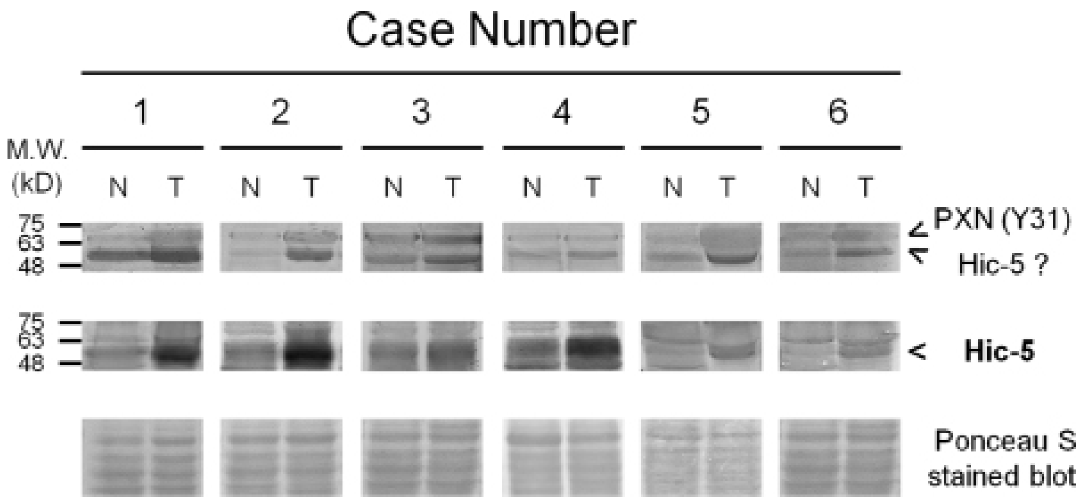

Supplementary Figure S1: Detection of Hic-5 and Tyr31-phosphorylated paxillin in HCC tissues. Western blot of Tyr31-phosphorylated paxillin [PXN(p-Y31)] (upper panel) and Hic-5 (middle panel) in tissue lysates of HCC from indicated patients using ponceau S stain as loading control. The data was representative of 3 reproducible experiments. N and T represent non-tumor and tumor sample respectively. The locations of indicated molecular weight marker are shown on the right. The question mark on the right indicated the suspected location of Hic-5 cross-reacting with Ab of PXN(p-Y31).

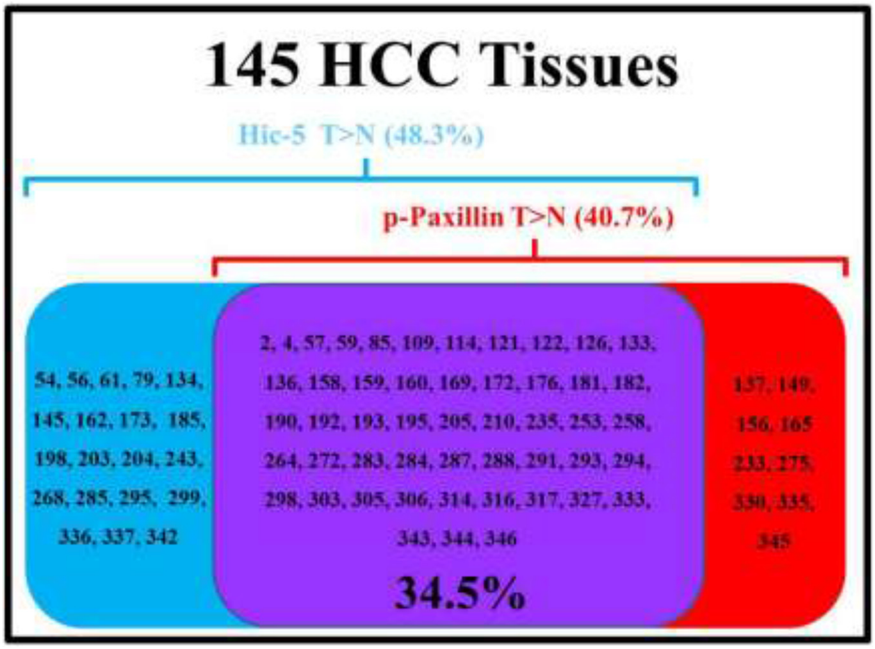

Supplementary Figure S2: proportions of HCCs with increased expression of Hic-5 and/or Tyr31-phosphorylated paxillin. The expression of Tyr31-phosphorylated paxillin (p-paxillin) and Hic-5 in tissue lysates of 145 HCCs from indicated patients (denoted as numbers) were examined by Western blot as described in Figure 1. The proportion of the HCCs with 2-fold higher p-paxillin and Hic-5 in tumor tissue compared with those in the normal counter parts ($T > N$) were calculated as 40.7% and 48.3%, respectively. The patients (denoted as numbers) with increased p-paxillin and Hic-5 are included in the area indicated by red and light blue brackets, respectively. The patient numbers belong to HCCs with simultaneous elevation of p-paxillin and Hic-5 (34.5%) are included with the purple area.

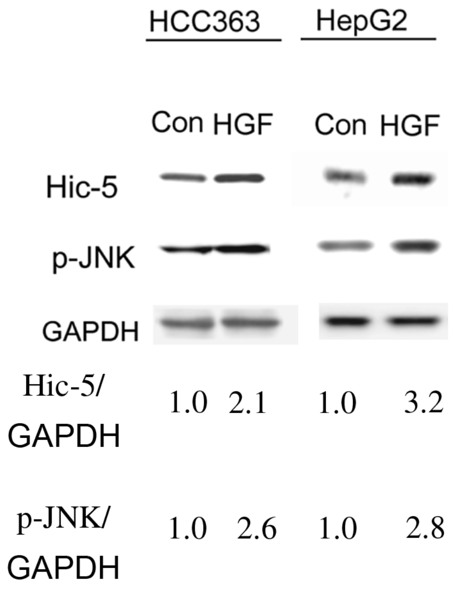

Supplementary Figure S3: HGF induced Hic-5 expression and JNK phosphorylation in H363 and HepG2. HCC363 and HepG2 were untreated (Con), treated with HGF for 24 h. Western blot of Hic-5 and phosphorylated JNK (p-JNK) were performed. GAPDH was used as loading control for the Western blots. The numbers shown below were averaged ($N = 2$) relative intensity ratio of Hic-5/GAPDH and p-JNK/GAPDH, taking the data of untreated (Con) as 1.0.

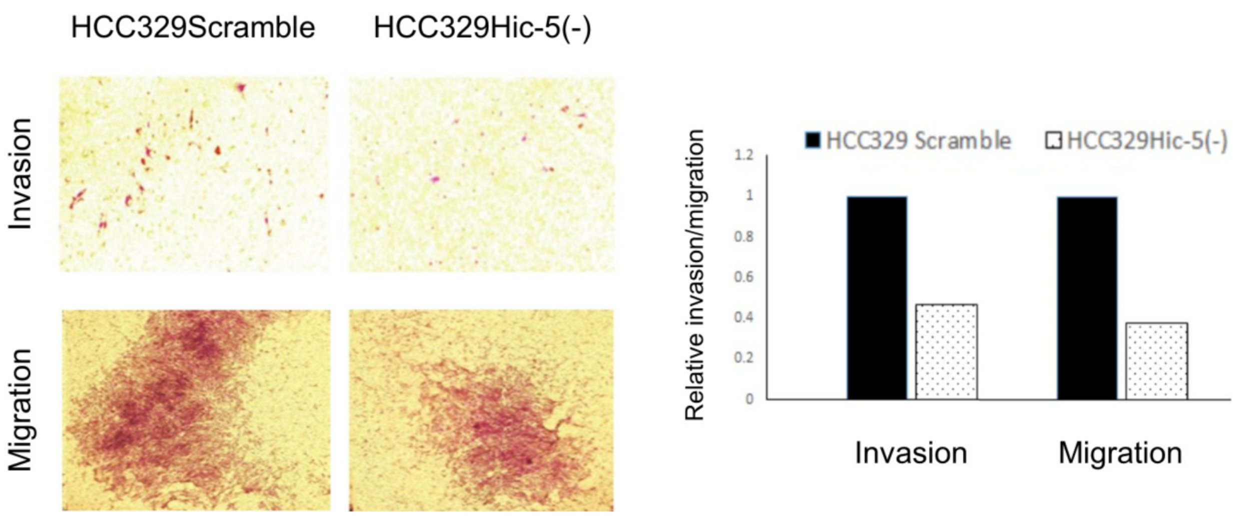

Supplementary Figure S4: Depletion of Hic-5 reduced cell migration and invasion of HCC329. HCC329Scramble and HCC329Hic-5(-) were seeded on matri-gel coated (left upper panel) or uncoated (left lower panel) cultured insert, cell invasion assay (left upper panel) and transwell migration assay (left lower panel) were performed. Quantitative data (from average of two results) was shown on the right panel. Relative invasion and migration were calculated taking the data of HCC329Scramble as 1.0.

(A)

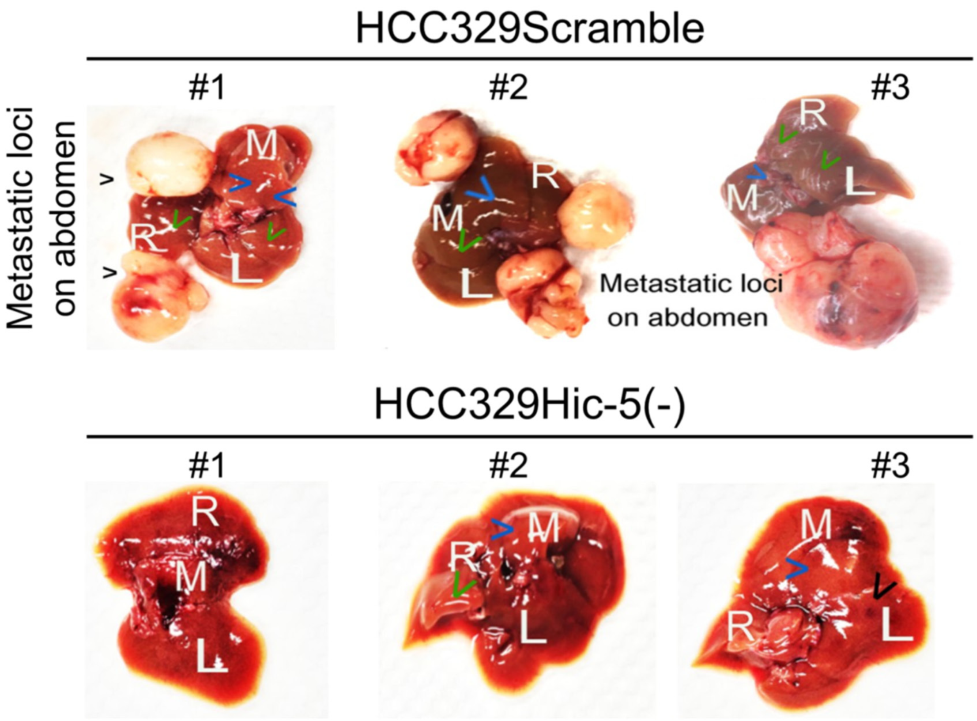

(B)

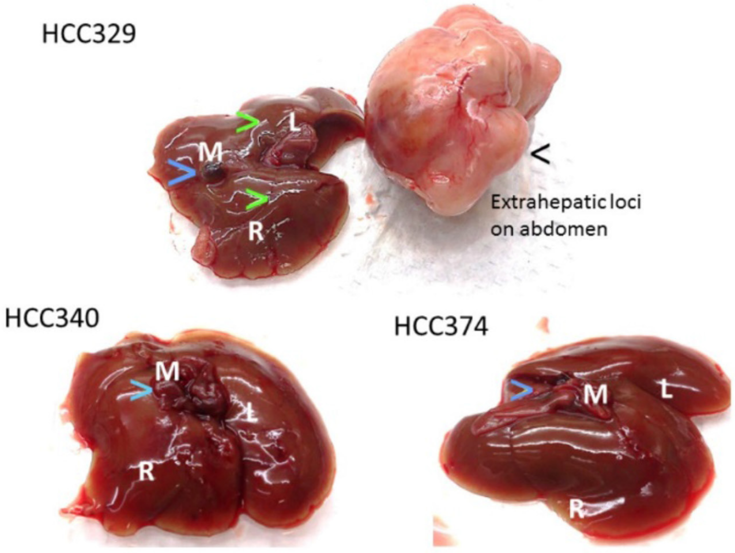

Supplementary Figure S5: Hic-5 is essential for HCC progression *in vivo*. Pictures of whole liver of SCID mice sacrificed after injection of each of 20 × 10^5 HCC329Scramble (upper) and HCCHic-5(-) **A.** and parental cell of indicated HCCs **B.** into middle lobe of liver for 2 months. The white letter M, L, R represent middle, left and right liver lobes respectively. "Blue" and "green" arrow heads indicated the location of primary and second tumors, respectively, in middle and right liver lobes. The round and white tumors shown in (A) and in (B) were obtained from an extrahepatic metastatic lesion in abdomen.

(A)

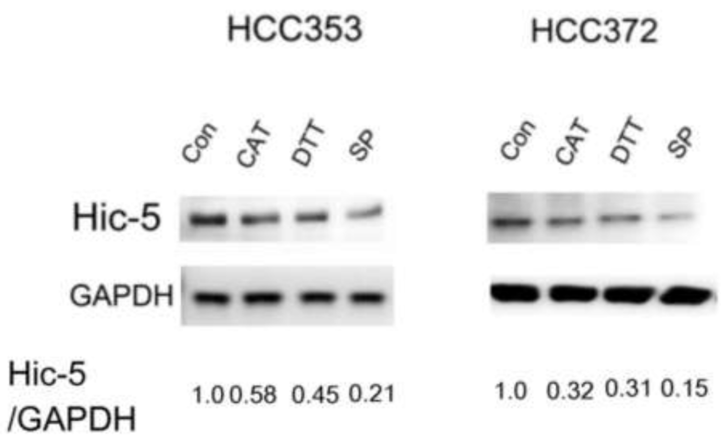

(B)

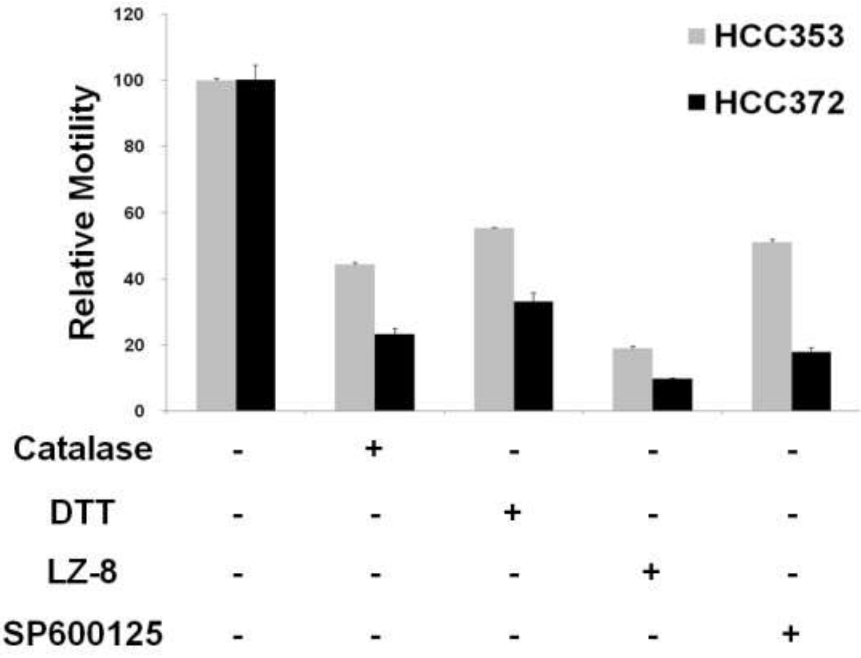

Supplementary Figure S6: ROS scavenger and JNK inhibitor suppressed Hic-5 expression and cell migration of HCC353 and HCC372. A. HCC353 (left panel) and HCC372 (right panel) were treated with 500 unit/ml catalase (CAT) or 0.5 mM dithiotheritol (DTT) for 24 h. Western blot of Hic-5 was performed, taking GAPDH as internal control. The numbers shown below were averaged ($N = 2$) relative intensity ratio of Hic-5/GAPDH. **B.** HCC353 and HCC372 were treated with catalase (CAT), dithiotheritol (DTT), LZ8 or SP600125 for 48 h. Wound healing motility assay were performed. Relative motility was calculated, taking the data of untreated cell as 1.0. The data shown are average from 2 reproducible experiments.

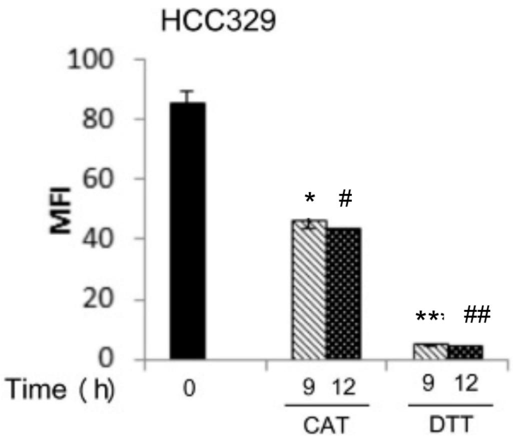

Supplementary Figure S7: The ROS scavenger catalase and dithiotheritol decreased ROS generation in HCC329. HCC329 were treated with 500 units/ml catalase (CAT) 0.5 mM dithiotheritol (DTT) at indicated time. ROS assay were performed. MFI: mean fluorescence intensity represent the G mean of DCF fluorescence detected in flow cytometry. (**)(##) and (*)(#) represent statistical significance ($p < 0.005$ and $p < 0.05$, respectively, $n = 4$) for differences of MFI between the indicated sample and time zero group.

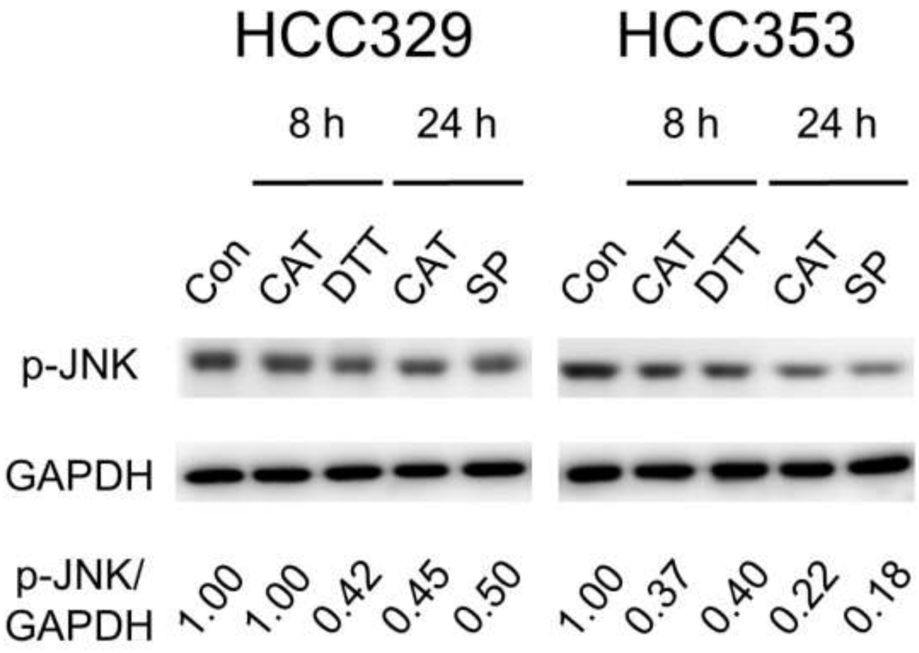

Supplementary Figure S8: ROS scavengers suppressed JNK phosphorylation of HCC329 and HCC353 in a time dependent manner. HCC329 (left panel) and HCC353 (right panel) were treated with 500 unit/ml catalase (CAT), 0.5 mM dithiotheritol (DTT) or 20 µM SP600125 (SP) for 8 and 24 h. Western blot of phosphorylated JNK (p-JNK) was performed, taking GAPDH as internal control. The numbers shown below were averaged ($N = 2$) relative intensity ratio of p-JNK /GAPDH.

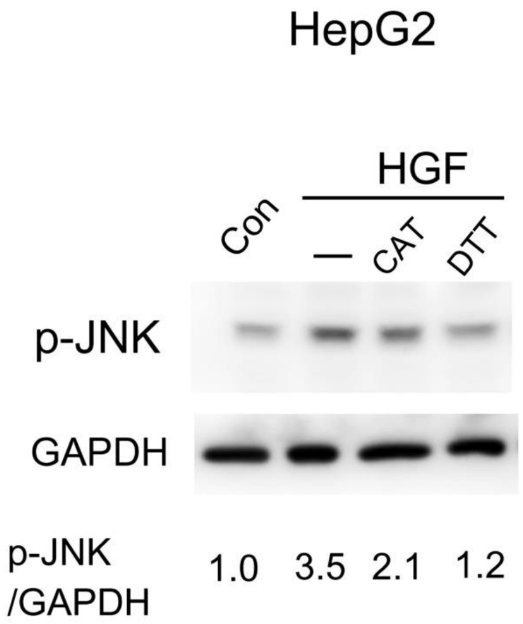

Supplementary Figure S9: ROS is essential for HGF-induced Hic-5 expression in HepG2. HepG2 cells were untreated (Con), treated with HGF, or HGF coupled with treated with 500 unit/ml catalase (CAT) or 0.5 mM dithiotheritol (DTT) for 24 h. Western blot of phosphorylated JNK (p-JNK) was performed using GAPDH as an internal control. The numbers shown below were averaged ($N = 2$) relative intensity ratio of p-JNK/GAPDH, taking the data of untreated (Con) as 1.0.

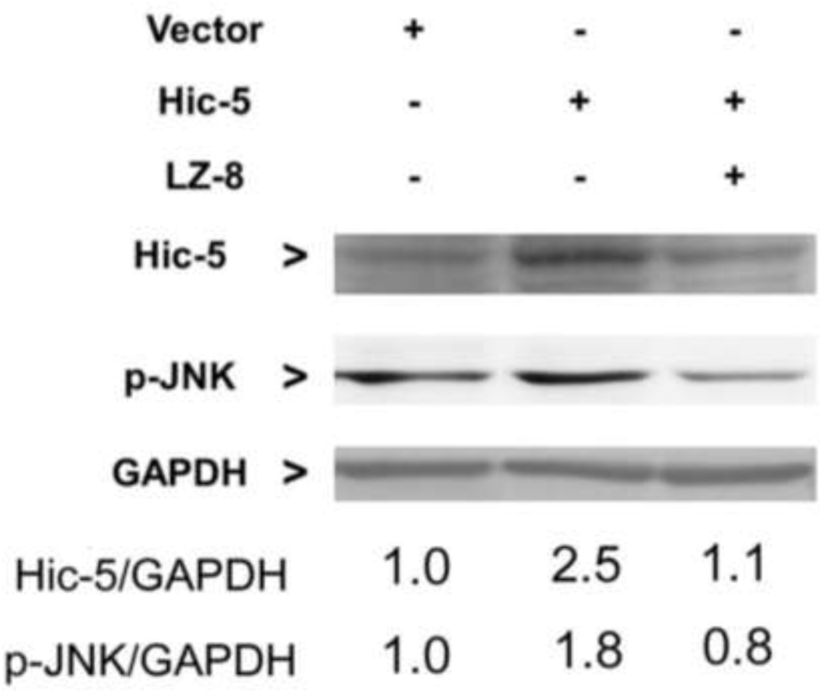

Supplementary Figure S10: overexpression of Hic-5 enhanced JNK phosphorylation which was suppressed by LZ-8. HCC340 was untransfected (MOCK), transfected with GFP control vector or Hic-5 overexpressing plasmid TGFB1I1 for 30 h, followed by treatment with none or LZ8 for 24 h. Western blot of Hic-5 and p-JNK was performed, taking GAPDH as an internal control. The numbers shown below were averaged ($N = 2$) relative intensity ratio of Hic-5/GAPDH and p-JNK /GAPDH.
